# Supplementary material for: Intermediate Repeat Expansion in the ATXN2 Gene as a Risk Factor in the ALS and FTD Spanish Population
Source: Biomedicines. 2024 Feb 2;12(2):356. doi: 10.3390/biomedicines12020356 (PMC10886453; doi:10.3390/biomedicines12020356)
Supplement: Supplementary file 1 [file biomedicines-12-00356-s001.zip › biomedicines-2809108-supplementary.pdf]

Supplementary Table S1. PCR amplification primers for *ATXN2*.

Primer	PCR	Sequence
SCA2-F	Standard	FAM-CGTGCGAGCCGGTGTATGGG
SCA2-R	Standard & RP-PCR	GGCCGACGCTAGAAGGCCGCT
AF-P3	RP-PCR	TACGCATCCCAGTTTGAGACG
SCA-P4	RP-PCR	FAM-TACGCATCCCAGTTTGAGCAGCAGCAGCAGCAGCAGCAGCAG

Supplementary Table S2. Standard PCR.

Reactive	Volume
Buffer 10X	2.5
dNTPs 1.25 mM	4
GC Rich	5
$MgCl_2$	1.5
DMSO	2.5
H_2O	4
Primer F	1.5
Primer R	1.5
FastStart Taq	0.5
ADN	2
MIX	23

* Volumes expressed in microliters (μL).

Supplementary Table S3. Repeat-primed PCR.

Reactive	Volume
Buffer 10X	2.5
dNTPs 1.25 mM	4
GC Rich	5
$MgCl_2$	1,5
H_2O	1,6
Primer R	2
AF-P3	2
SCA-P4	2
Taq FastStart	0,4
ADN	4

* Volumes expressed in microliters (μL).
